# Impaired epidermal Langerhans cell maturation in TGFβ-inducible early gene 1 (TIEG1) knockout mice

**DOI:** 10.18632/oncotarget.22843

**Published:** 2017-12-01

**Authors:** Xilin Zhang, Yi Yao, Wei-Zen Wei, Zeng-Quan Yang, Jun Gu, Li Zhou

**Affiliations:** ^1^ Center for Cutaneous Biology and Immunology Research, Department of Dermatology, Henry Ford Health System, Detroit, MI, USA; ^2^ Immunology Research Program, Henry Ford Cancer Institute, Henry Ford Health System, Detroit, MI, USA; ^3^ Department of Dermatology, Second Military Medical University Changhai Hospital, Shanghai, China; ^4^ Karmanos Cancer Institute, Wayne State University, Detroit, MI, USA; ^5^ Department of Internal Medicine, Henry Ford Health System, Detroit, MI, USA

**Keywords:** Langerhans cell, TIEG1, maturation, TGF-β1 signaling

## Abstract

TGF-β-inducible early gene 1 (TIEG1), also known as Krüppel-like factor 10 (Klf10), represents a major downstream transcription factor of transforming growth factor-β1 (TGF-β1) signaling. Epidermal Langerhans cells (LCs), a unique subpopulation of dendritic cells (DC), essentially mediates immune surveillance and tolerance. TGF-β1 plays a pivotal role in LC maintenance and function after birth, although the underpinning mechanisms remain elusive. Here, we hypothesized that TIEG1 might be involved in TGF-β1-mediated LC homeostasis and function. Utilizing TIEG1 null mice, we discovered that TIEG1 deficiency did not alter LC homeostasis at the steady state and LC repopulation at inflamed-state, as well as their antigen-uptake capacity, but significantly impaired their maturation ability, which was opposite to the fact that loss of TGF-β1 induced spontaneous LC maturation. Moreover, the ablation of TIEG1 enhanced skin contact hypersensitivity response. Our results suggested that TIEG1 is not a key molecule involved in TGF-β1-mediated homeostasis, while TIEG1-related signaling pathways regulate LC maturation and their function.

## INTRODUCTION

Epidermal Langerhans cells (LCs) represents an exclusive subpopulation of skin-resident dendritic cells (DCs), which characteristically express the C-type lectin receptor Langerin (CD207) and its associated “tennis-racket” organelle, Birbeck granules [1]. Adult mouse LCs largely derive from embryonic fetal liver monocytes, with a small minority originate from yolk-sac macrophages [2]. They slowly self-renew *in situ* under steady-state whereas being supplemented by hematopoietic precursors in inflamed-state [3, 4]. Upon the capture of external or internal antigens, immature LCs leave the epidermis and migrate to nearby draining lymph nodes (LNs) where they turn into mature counterparts. Subsequently, these mature LCs induce T cell immune responses by presenting engulfed antigens to naïve T cells along with secreting cytokines for their differentiation as well as providing costimulatory signals [5]. The aberrant expression of cell-membrane costimulatory molecules on LCs has been implicated in multiple skin disorders including contact hypersensitivity, atopic dermatitis, and virus infection [6–8]. Hence, the elaboration of decisive factors during the maturation of epidermal LCs are of potential therapeutic benefit.

Transforming growth factor-β1 (TGF-β1), an important immunomodulatory and profibrotic cytokine, essentially regulates the maintenance of epidermal LCs after birth [9]. TGF-β1 constitutive null mice lacked LCs [10]. Moreover, LC-specific excision of TGF-β1 or TGF-β receptor II (TGFβRII) resulted in a profound LC loss, suggesting a direct impact of TGF-β1 on LCs through an autocrine or paracrine loop [11]. Further analysis with inducible depletion of TGF-β1 signaling in adult LCs demonstrated a crucial role of TGF-β1 in suppressing LC migration as well as preventing their spontaneous maturation [12–14]. However, the underpinning mechanism of TGF-β1-directed LC biology remained largely unknown. We have recently identified that neither Smad2, Smad3 nor Smad4 in canonical TGF-β1-Smad pathway was required for LC homeostasis [15, 16]. So far, only a limited number of TGF-β1-relevant transcription factors mediating LC development and maintenance have been reported [9].

TGFβ-inducible early gene 1 (TIEG1), also known as Krüppel-like factor 10 (Klf10), was originally discovered in normal human osteoblast cells and termed for its rapid induction by TGF-β1 without *de novo* protein synthesis [17]. Sequence analyses demonstrated that the evolutionarily-conserved TIEG1 gene encodes a 480 amino-acid protein comprised of a three-zinc finger motif, a Sp1-like DNA binding domain and multiple proline-rich Src homology-3 (SH3) binding domains [17, 18]. TIEG1 enhanced TGF-β1/Smad signaling by the repression of Smad7 gene and induction of Smad2 gene expression [19]. Later studies discovered that TIEG1 expression could be induced by a plethora of growth factors, cytokines, and hormones, which served as a potential marker for skeletal, heart and neoplastic diseases [17, 19–22]. However, the role of TIEG1 in TGF-β1-mediated LC homeostasis remains unexplored.

In this study, we sought to address the effect of TIEG1 deficiency on epidermal LC homeostasis, phagocytosis, maturation *in vitro*, and cell repopulation under inflamed condition. For the first time, we demonstrated that the ablation of TIEG1 did not hamper the survival of LCs in steady-state, cell replenishment under inflamed-state nor their capability of antigen uptake, but specifically prohibited their maturation.

## RESULTS

### TIEG1 is not required for epidermal LC homeostasis under the steady state

**Figure 1:**
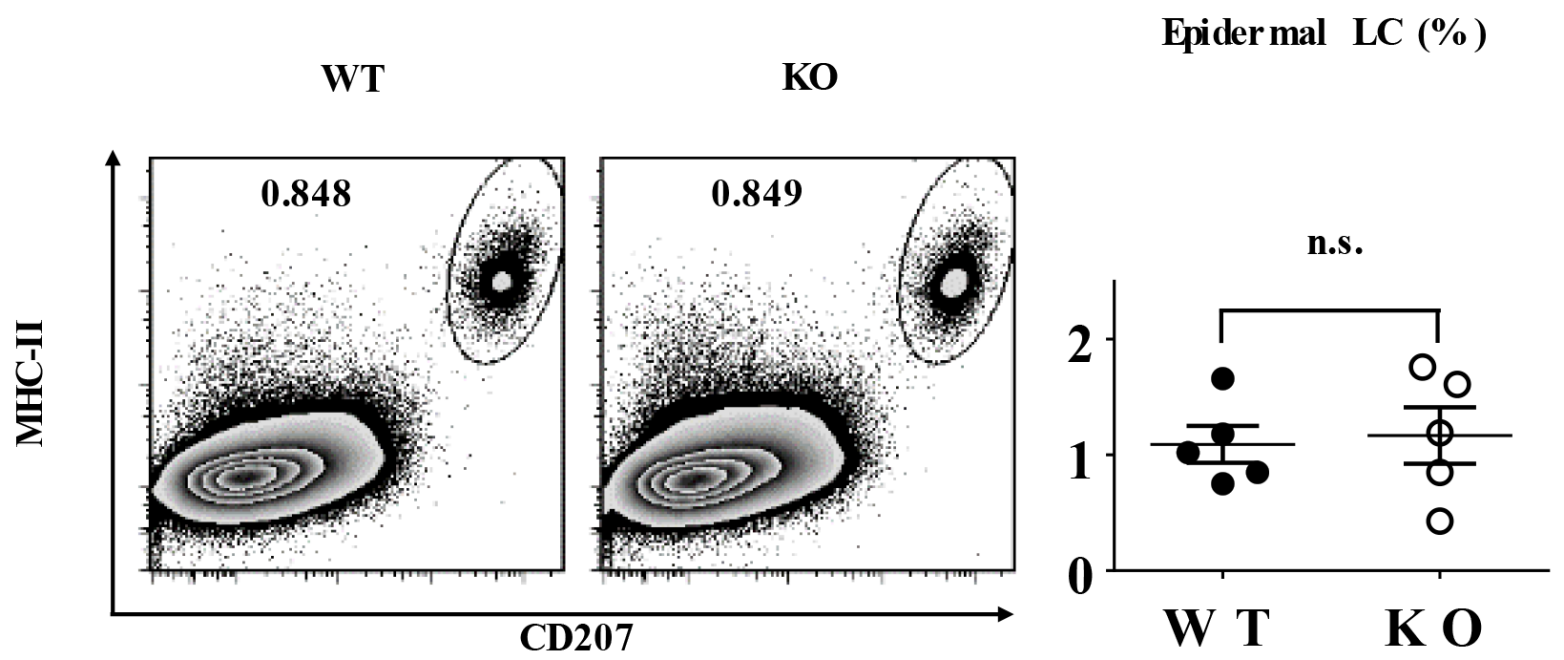
TIEG1 deficiency did not affect LC homeostasis Epidermal cells were freshly-isolated from the trunk skin of TIEG1 knockout (KO) and wild-type C57BL/6 (WT) mice. Representative FACS analysis (left panel) and the ratios (right panel) of epidermal LCs from TIEG1 WT and KO mice (n=10, three independent experiments).

To investigate a possible regulatory role of TIEG1 in LC homeostasis, we first compared the epidermal LC ratios of TIEG1^-/-^ C57/BL6 mice (designated as KO mice) to wild-type C57/BL6 mice (designated as WT mice), and found that the deletion of TIEG1 did not alter the density of epidermal LCs (Figure 1). Thus, our result suggested that unlike TGF-β1, TIEG1 is not required for LC homeostasis under the steady state.

### TIEG1 deficiency does not hamper LC phagocytosis

**Figure 2:**
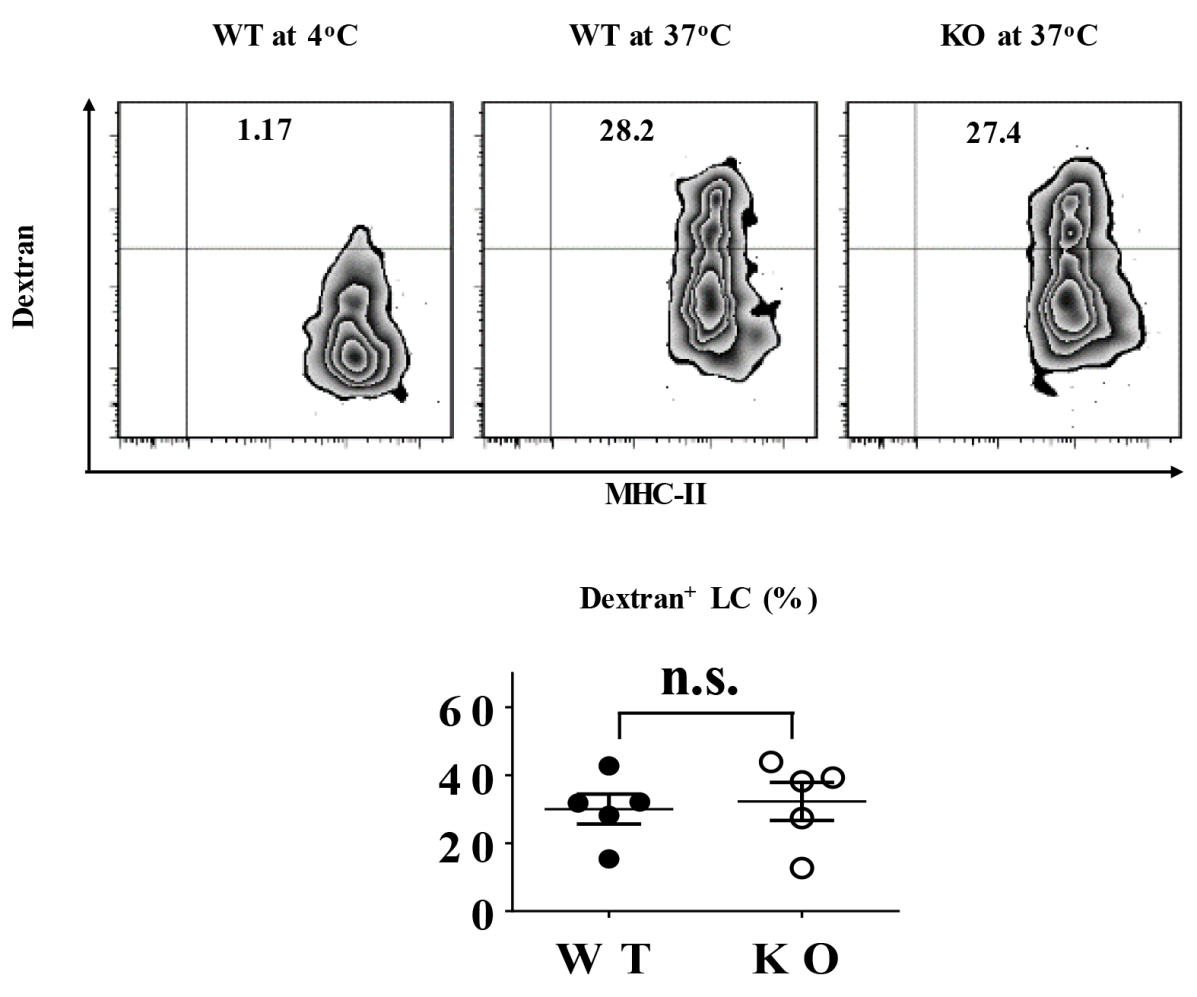
TIEG1 was not required for LC phagocytosis Freshly-isolated epidermal LCs from TIEG1 WT and KO mice were incubated at 37°C or 4°C (as control) with Dextran-FITC for 45 minutes. Representative FACS analysis (upper panel) and the percentages (lower panel) of FITC^+^ epidermal LCs (n=10, two independent experiments).

Typically, immature LCs capture and process external or internal antigens, migrate to the nearby draining lymph nodes where they fulfill maturation, present phagocytosed antigens to naïve T cells and subsequently induce T cell priming. Thus, the phagocytizing capacity of LCs is crucial for their immunoregulatory function. To evaluate the role of TIEG1 in LC antigen-uptake, freshly-isolated epidermal cells from TIEG1 WT and KO mice were incubated with dextran-FITC for 45 minutes at 37 °C or 4 °C (as control). The percentages of dextran-FITC^+^ LCs were comparable between TIEG1 WT and KO mice (Figure 2), implying that lack of TIEG1 did not affect the phagocytizing ability of LCs.

### Lack of TIEG1 prohibits LC maturation

**Figure 3:**
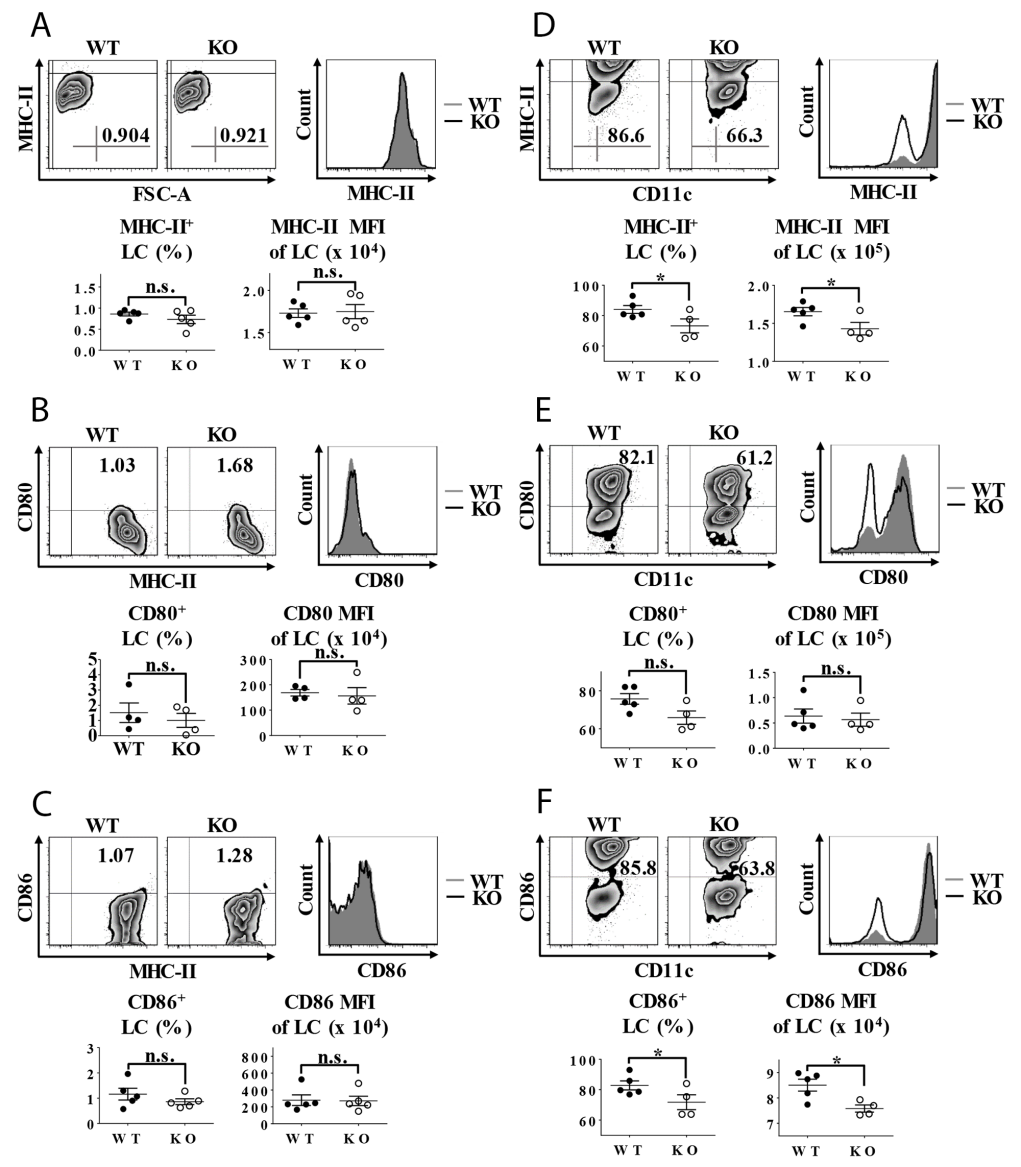
Impaired LC maturation in TIEG1 knockout mice **(A)** Epidermal suspensions freshly isolated from the trunk skin were stained with anti-MHC-II, CD45.2, CD80, and CD86 antibodies and analyzed by flow cytometry. Representative FACS analysis (upper-left panel), histogram (upper-right panel) and the ratios (lower panel) of MHC-II^+^ epidermal LCs from TIEG1 WT and KO mice (n=10, three independent experiments); **(B)** Epidermal cells were stained as described in (3a). Representative FACS analysis, histogram and the ratios of CD80^+^ epidermal LCs from TIEG1 WT and KO mice (n=8, two independent experiments); **(C)** Epidermal cells were stained as described in (3a). Representative FACS analysis, histogram and the ratios of CD86^+^ epidermal LCs from TIEG1 WT and KO mice (n=10, three independent experiments); **(D)** Epidermal suspensions from the trunk skin of TIEG1 WT and KO mice were cultured in RPMI for 60 hours, and then stained as described in (3a). Representative FACS analysis, histogram and the ratios of MHC-II^+^ epidermal LCs from TIEG1 WT and KO mice (n= 9, three independent experiments); **(E)** Epidermal cells were treated as in (3d). Representative FACS analysis, histogram and the ratios of CD80^+^ epidermal LCs from TIEG1 WT and KO mice (n=9, three independent experiments); **(F)** Epidermal cells were treated as in (3d). Representative FACS analysis, histogram and the ratios of CD86^+^ epidermal LCs from TIEG1 WT and KO mice (n=9, three independent experiments).

The maturation conversion of epidermal LCs is characteristically accompanied with an upregulated expression of cell-membrane costimulatory molecules, including major histocompatibility complex-II (MHC-II), CD80 and CD86. Under steady state, the baseline expression ratios of MHC-II, CD80 or CD86 and their mean fluorescence intensity (MFI) on the freshly-isolated epidermal LCs from TIEG1 WT and KO mice were equally low (Figure 3A–3C). However, after 60 hours of *in vitro* culture, the expression level of MHC-II, CD80 and CD86 in TIEG1-deficient LCs were considerably lower than their normal counterparts, although the difference in CD80 expression did not reach statistical significance (Figure 3D–3F). These results indicated that in contrast to TGF-β1, TIEG1 deficiency did not disturb the immature status of epidermal LCs but impaired their capacity to acquire a mature phenotype *in vitro*.

### TIEG1 deficiency enhanced contact hypersensitivity

**Figure 4:**
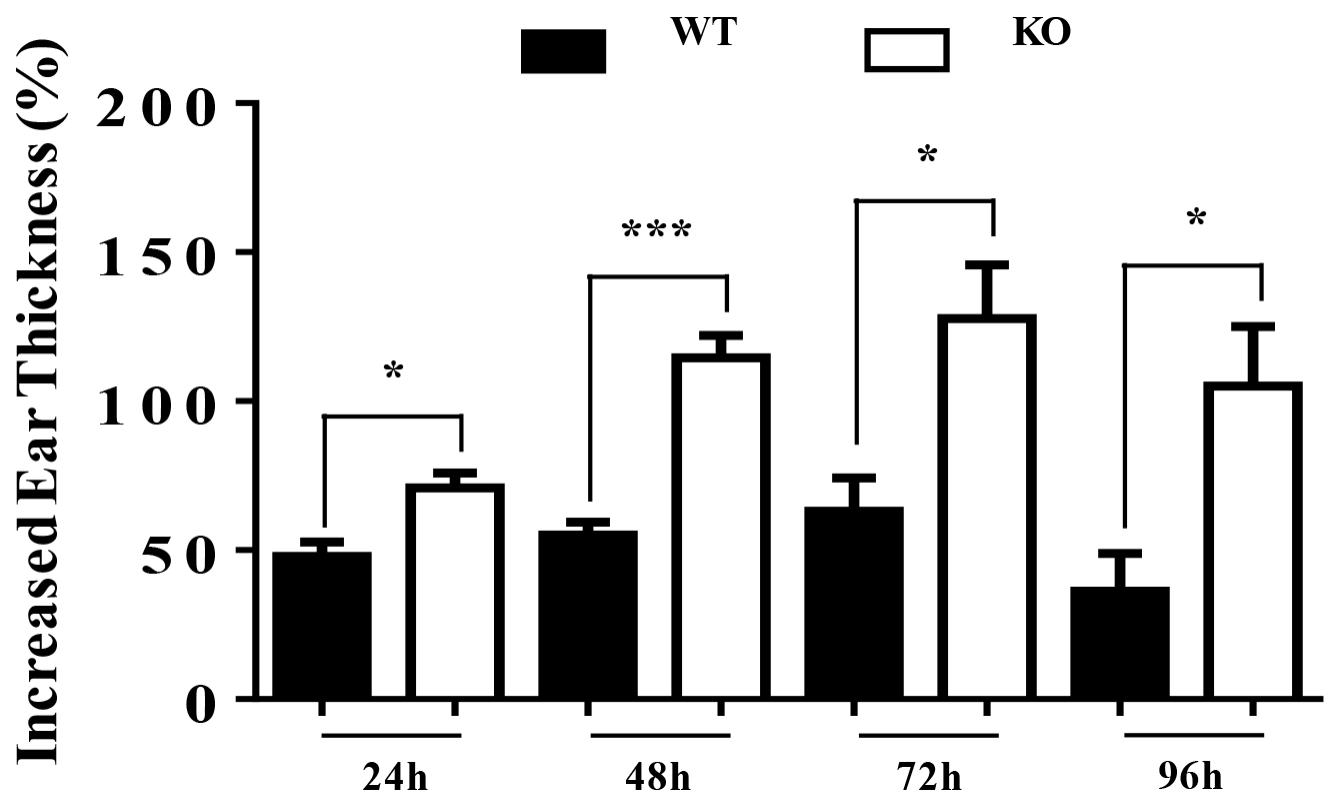
TIEG1 deficiency enhanced contact hypersensitivity response Mice were sensitized with DNFB (0.5%) in the abdominal area and then challenged with DNFB (0.2%) 5 days later to induce delayed type hypersensitivity in the ears. Ear thicknesses were measured at different time points after challenge (24 hours, 48 hours, 72 hours, 96 hours) by comparing challenged and unchallenged ears using a thickness gauge in a blinded manner. Ten mice were analyzed.

To assess the role of TIEG1 in contact hypersensitivity (CHS), we sensitized TIEG1 WT and KO mice by topically applying 0.5% dinitrofluorobenzene (DNFB) on abdominal skin, and then challenged with 0.2% DNFB five days later. The percentages of increased ear thickness caused by the challenge in TIEG1 KO mice were significantly higher than in TIEG1 WT mice at different time points (24 hours, 48 hours, 72 hours, 96 hours) (Figure 4). This result indicated that TIEG1 deficiency enhanced CHS response.

### TIEG1 is not involved in LC replenishment

**Figure 5:**
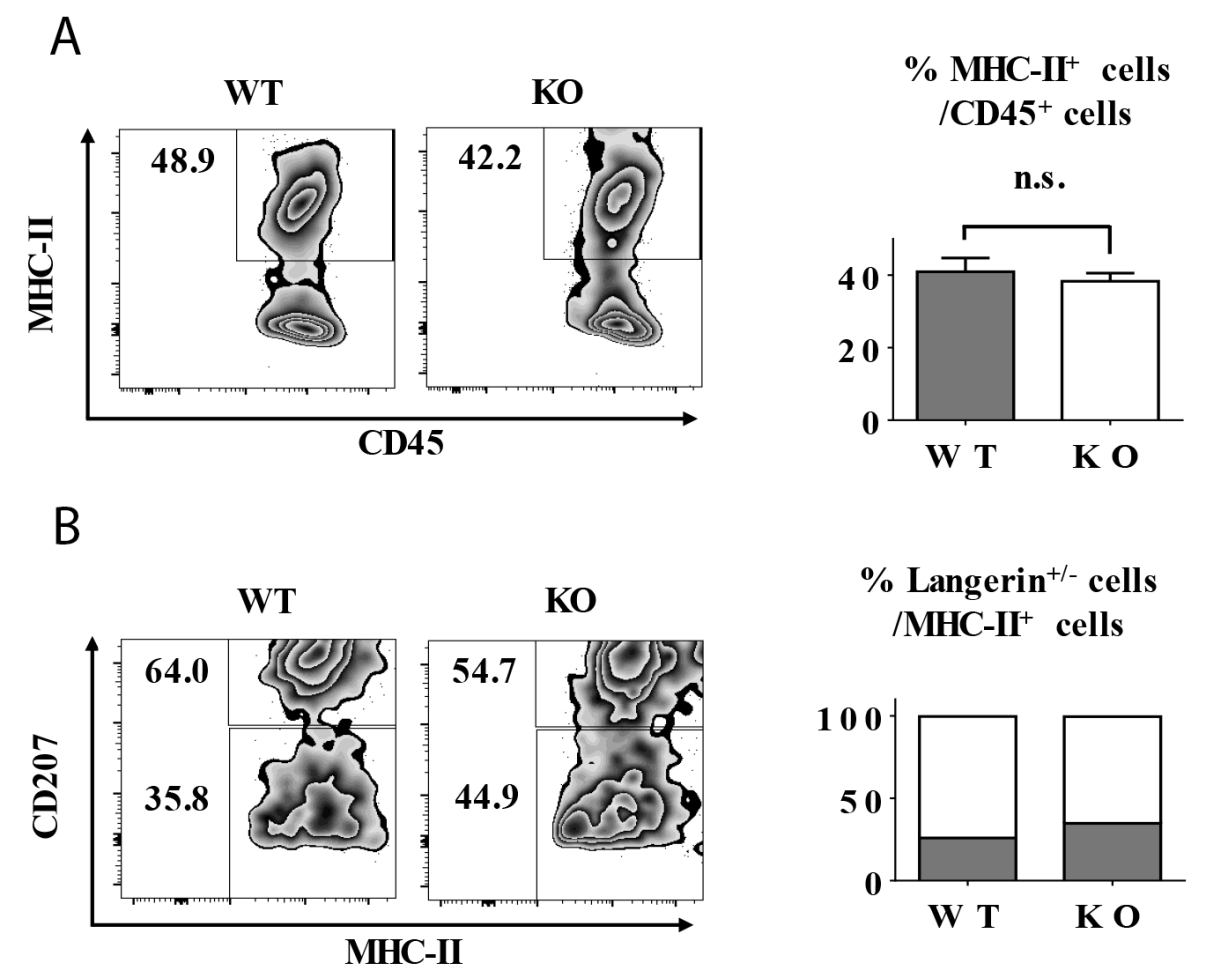
TIEG1 deficiency did not impair LC repopulation in inflamed state The mice were treated with 15 minutes of UV exposure. **(A)** Representative FACS analysis (left panel) and the ratios (right panel) of epidermal LCs (MHC-II^+^) one week after UV treatment. **(B)** Representative FACS analysis (left panel) and the frequencies (right panel) of short-term LCs (MHC-II^+^ langerin^low^) and long-term (MHC-II^+^ langerin^+^) cells from each genotype. The data represent the result of 12 mice.

To explore the role of TIEG1 in LC repopulation, we provoked skin inflammation in TIEG1 WT and KO mice by 15 minutes of ultraviolet (UV) irradiation. As expected, UV treatment caused a transient loss of LCs which were replenished by short-term LCs (MHC-II^+^ langerin^low^) (Figure 5) and long-term LCs (MHC-II^+^ langerin^+^) successively (data not shown) [4]. In detail, the epidermal LC ratios one week after UV irradiation were comparable between TIEG1 WT and KO mice (Figure 5A), and the percentages of short-term and long-term LCs were also not altered by TIEG1 deficiency (Figure 5B). Hence, these results implied that TIEG1 was not required for the repopulation of LCs under inflamed state.

## DISCUSSION

As one of the TGF-β1 down-stream transcription factors, TIEG1 enhanced TGF-β1 signaling by inhibiting Smad7 gene and promoting Smad2 gene expression [19]. Besides, TIEG1 could also directly increased TGFβRII gene expression through histone H3 acetylation [23]. Hence, TIEG1 might be a major positive regulator in TGF-β1 signaling. Previous studies suggested that TGF-β1 is a prerequisite for LC homeostasis and maintenance in skin, and TGF-β1 inhibits LC maturation and migration, but does not affect LC replenishment under inflamed-state [10–14]. Our results demonstrated that unlike TGF-β1, TIEG1 was not essential for LC homeostasis but is required for the full maturation of LCs. The discrepancy in LC phenotype between TGF-β1- and TIEG1-deficient mice suggested that TIEG1 may not be a key molecule involved in TGF-β1-directed LC homeostasis, while TIEG1-related signaling pathways modulate LC maturation. It has been reported that TIEG-1 overexpression leads to the activation of p38 MAPK, which could induces costimulatory molecule expression on the LCs and enhanced their T-cell stimulatory capacity [24–26]. Whether TIEG1 mediates the maturation process of LCs through p38 MAPK or other factors awaits future investigation.

Epidermal LCs were initially considered as solely immunogenic that determined contact hypersensitivity (CHS) [27]. Recent *in vivo* studies on LC-ablative mouse models uncovered that epidermal LCs might mainly play a tolerogenic role in CHS [28]. We demonstrated here that although the maturation status of LCs was compromised in TIEG1 null mice, they developed more severe CHS response than WT mice, which further verified that LCs principally mediated immune tolerance in CHS. Moreover, our finding was same as the augmented phthalic anhydride-induced skin inflammation in TIEG1 KO mice reported by Bae *et al* [26]. However, the exact role of LCs in TIEG1-mediated suppression of CHS response require further examination.

LC replenishment under inflamed condition is achieved by two waves of distinct LC populations: a minor transient population of short-term LCs (MHC-II^+^ langerin^low^) originated from circulating monocytes followed by a major persistent population of long-term LCs (MHC-II^+^ langerin^+^) of uncharacterized bone-marrow (BM) precursors [4]. Previous research demonstrated that lack of TGF-β1 exerted no impairment on the BM's potential to generate LCs *in vivo*, which suggested that TGF-β1 was probably not required for the LC repopulation in inflamed state [10]. The negligible role of TIEG1 in LC replenishment we identified in this study further supported this assumption.

In summary, our results indicate that TIEG1 is not involved in TGF-β1-directed LC homeostasis, but contrarily facilitates LC maturation. Additionally, TIEG1 deficiency enhanced DNFB-induced CHS response.

## MATERIALS AND METHODS

### Mice

TIEG1 KO mice have been described previously [29]. C57BL/6 (WT) mice were purchased from the Jackson Laboratory. Experiments were conducted at 7 to 9 weeks of age. Mice were housed in a specific pathogen-free barrier unit. Handling of mice and experimental procedures were in accordance with the requirements of Institutional Animal Care and Use Committee.

### Skin single-cell suspension preparations

The trunk skin of TIEG1 WT and KO mice was incubated in 0.5% dispase (Gibco, Life Technologies, Grand Island, NY, USA) for 1 hour at 37°C after its subcutaneous fat was scraped off. Then, epidermal sheet was peeled from the dermis, cut into tiny pieces and digested in complete culture medium containing 0.01% DNase (Sigma, St Louis, MO, USA) for 1 hour at 37°C. The epidermal single-cell suspensions were harvested after filtering through a 70 μM filter. Complete culture medium was RPMI 1640 (with 2mM L-glutamine, Gibco) supplemented with 10% heat-inactivated fetal bovine serum (Hyclone, Thermo Scientific, Pittsburgh, PA, USA), 5 × 10^5^ M 2-mercaptoethanol, 0.15% sodium hydrogencarbonate, 1 mM sodium pyruvate, nonessential amino acids, 100U per ml penicillin and 0.01% streptomycin.

### Flow cytometry and antibodies

Single-cell suspensions were pretreated with anti-FcγRII/III (clone 2.4G2) for 10 mins at 4°C, and then stained for surface and intracellular markers with the conjugated monoclonal antibodies listed as below: MHC-II (I-A/E) (M5/114.15.2), CD45.2 (104), CD11c (N418), CD80 (16-10A1), CD86 (GL1) and CD207 (929F3.01). All antibodies were purchased from eBioscience (San Diego, CA, USA) or Dendritics (Lyon, France). Cells were analyzed with FACS Aria II (BD Biosciences, San Jose, CA, USA) and FlowJo software version 7.6.1 for Microsoft (TreeStar, Sam Carlos, CA, USA).

### Phagocytosis assay

Freshly-isolated epidermal cells were incubated with 0.025% Dextran-Fluorescein Isothiocyanate (FITC) (Life Technologies, Grand Island, NY, USA) for 45 mins at 37°C or 4°C. Then, cells were washed by phosphate buffered saline (PBS), and subsequently stained with anti-CD45.2 and MHC-II, the percentage of LCs that uptake antigen (CD45.2^+^/MHC-II^+^/FITC^+^) was determined by flow cytometry.

### *In vitro* maturation

Freshly-isolated epidermal cells were cultured with complete culture medium at 37°C for 60 hours. The cells were then collected and stained with anti-MHC-II, CD207, CD45.2, CD80 and CD86 for flow cytometric analysis.

### Hapten sensitization and elicitation of CHS

On day 0, mice were sensitized by applying 25μl of 0.5% DNFB (Sigma-Aldrich, St. Louis, MO, USA) (acetone:olive oil = 4:1) on shaved belly skin. On day 5, sensitized mice were challenged topically with 10μl of 0.2% DNFB on the left ear, whereas 10μl of acetone/olive oil (4:1) was painted on the right ear. Ear thicknesses were measured by comparing challenged (left) and unchallenged (right) ears using a thickness gauge (Digimatic caliper, Mitutoyo, Japan) in a blinded manner. And, ear thickness increases were calculated by subtracting pre-challenge (0 hour) from post-challenge measurements (24 hours, 48 hours, 72 hours, 96 hours).

### LC replenishment under skin inflammation

For the depletion of LCs, mice were exposed to ultraviolet (UV) for 15 minutes (wavelength 254 nm, voltage 8W, source: 38 cm). The replenishment of the LC network was assessed 1 week after UV treatment.

### Statistical analysis

Data were presented as mean ± standard deviation (SD). Statistics analyses were performed with GraphPad Prism software (GraphPad, San Diego, CA, USA) using a two-tailed Student t test. Differences were considered to be statistically significant when P < 0.05.
